# Epilepsy surgery in patient with monogenic epilepsy related to SCN8A mutation

**DOI:** 10.1016/j.ebr.2022.100536

**Published:** 2022-03-22

**Authors:** Irina Podkorytova, Ryan Hays, Ghazala Perven, Sasha Alick Lindstrom

**Affiliations:** Department of Neurology, University of Texas Southwestern Medical Center, 5323 Harry Hines Blvd, Dallas, TX 75390-8508, United States

**Keywords:** FBTC, focal to bilateral tonic-clonic, SEEG, stereoelectroencephalography, SPECT, single-photon emission computerized tomography, VUS, variant of uncertain significanse, Epilepsy surgery, Genetic testing, SCN8A mutation, Stereoelectroencephalography

## Abstract

Epilepsy surgery is superior to prolonged medical therapy in patients with drug-resistant focal epilepsy, but reports on epilepsy surgery outcomes for patients with a genetic etiology are limited, especially in adults. This is the first documented report of a stereoelectroencephalography (SEEG) evaluation and resective surgery outcome in an adult patient with epilepsy related to SCN8A mutation.

We describe a patient with epilepsy related to SCN8A mutation which was reported as a variant of uncertain significance at time of his pre-surgical evaluation and reclassified as likely pathogenic about 3 years after resective epilepsy surgery. Most of his pre-surgical evaluation results suggested right temporal lobe epilepsy, but few reported semiological symptoms, ictal SPECT, and neuropsychology results were discordant, and brain MRI was non-lesional. Therefore, SEEG was recommended; ultimately, seizures were localized to the right hippocampus. He was seizure-free for 1.5 years after right anterior temporal lobectomy, then reported three focal to bilateral tonic-clonic (FBTC) seizures in the subsequent 12 months (preoperatively, 6 focal impaired awareness seizures and 4–6 FBTC per year).

This case demonstrates that epilepsy surgery reduced seizure burden in a patient with SCN8A-related epilepsy granting him short-term seizure freedom after resection, and then decreased seizure frequency after relapse compared to the preoperative baseline.

## Highlights

• This is the first epilepsy surgery report in patient with SCN8A mutation.
• Stereo-EEG evaluation localized seizure onset to the right hippocampus.
• Resection led to 1.5-year seizure freedom, then seizures relapsed.
• Seizure frequency after relapse was significantly lower than preoperatively.
• Epilepsy surgery reduced seizure burden in patient with SCN8A-related epilepsy.

## 1 Introduction

Epilepsy surgery is superior to prolonged medical therapy in patients with drug-resistant focal epilepsy, but reports on epilepsy surgery outcome for patients with genetic drug-resistant epilepsy are limited, especially in adults [1], [2], [3].

We aim to describe the stereo-EEG (SEEG) evaluation results and resective surgery outcome in a patient with epilepsy related to SCN8A mutation (c.2671G > A, p.Val891Met, heterozygous) which was initially reported as a variant of uncertain significance (VUS) at time of his pre-surgical evaluation, but later reclassified as likely pathogenic 3 years after his SEEG monitoring (2 years 10 months after resective epilepsy surgery).

## 2 Case presentation

At time of the first pre-surgical presentation, the patient was 24 years old. He is a right-handed Caucasian man with his first seizure occurring in his first week of life. He had some degree of seizure control and was even able to wean off anti-seizure medications (ASM) at various points throughout childhood and adolescence. But, by age 18-years-old, seizures became drug-resistant despite trials of 8 ASM and vagus nerve stimulation (VNS).

Our patient endorsed a family history of epilepsy. His father had a personal history of focal to bilateral tonic-clonic seizures (FBTC) and status epilepticus, but he was well controlled on valproate. The patient’s older brother had FBTC since 22 months of age. He was treated with carbamazepine, but his seizures were not well controlled. He passed away at the age of 20 years, reportedly due to myocardial infarction during a seizure. The autopsy found previously unidentified hypertrophic cardiomyopathy. The fraternal twin brother of our patient does not have epilepsy.

At the time of the pre-surgical evaluation, our patient was experiencing focal impaired awareness seizures every two months described as behavioral arrest with unresponsiveness and oral automatisms followed by versive head turn reportedly to the right (laterality based on historical recall by the family). Four to six times per year focal seizures progressed to bilateral tonic-clonic seizures accompanied by urinary incontinence and tongue bite. His seizures usually happened at night upon going to sleep or within an hour of waking up in the morning, and often occurred in clusters. Self-reported triggers included missed or delayed medications, sleep deprivation and stress. He had a history of status epilepticus during video-EEG monitoring in an outside hospital when all of his ASM were stopped on day one of admission. The patient also historically reported somatosensory involving his right arm and olfactory or auditory auras which were not documented during his admission to our epilepsy monitoring unit (EMU). His neurological examination was unremarkable.

During his pre-surgical evaluation at our center, five focal impaired awareness seizures with oral automatisms were recorded, and one event progressed to a FBTC seizure preceded by versive head turn to the left. On scalp EEG, these electroclinical seizures were localized to the right anterior temporal region (FT10 maximum), and the interictal EEG also demonstrated intermittent theta slowing in the right temporal region. His brain MRI was normal. PET showed right anterior mesial temporal hypometabolism, while the ictal single photon emission computed tomography (SPECT) demonstrated right anterior lateral temporal hyperperfusion. The neuropsychological evaluation noted dysfunction of nondominant hemisphere systems, and also impaired processing speed and slow performance across virtually all measures. His full-scale intelligence quotient (FSIQ) was in the low average range. The functional MRI localized his language bilaterally with right hemisphere dominance, and the Wada test demonstrated bilateral language and memory. An autoimmune epilepsy antibody panel was negative. An epilepsy gene panel (Invitae, San Francisco, CA; sequence analysis and deletion and duplication testing of the 181 genes) at the time of pre-surgical evaluation identified four variants of uncertain significance (VUS) in CACNA2D2, SCN8A, SYNJ1 and SZT2; therefore, his genetic testing was deemed not conclusive for a monogenic epilepsy disorder at that time.

Although most of his pre-surgical evaluation results suggested right temporal lobe epilepsy, his MRI-brain was non-lesional and the surgical committee advised that further intracranial video-EEG monitoring was necessary to determine the seizure-onset zone. This was due to historical reports of versive head turn in the opposite direction than what was recorded on scalp ictal EEG. In addition, there were historical reports of auditory and somatosensory auras and also historical reports of possible auditory and somatosensory auras, as well as a history of frequent FBTC which would be less typical for mesial temporal epilepsy. Furthermore, ictal SPECT suggested a more lateral temporal neocortical onset. The committee recommended SEEG as the optimal approach to further localize the seizure onset zone(s). The SEEG coverage was designed to sample mesial and lateral temporal structures in the right temporal lobe, temporal lobe epilepsy mimics in the right hemisphere, and mesial temporal structures in the contralateral temporal lobe to exclude independent bilateral temporal, temporal-plus and extra-temporal seizure focus [4], [5].

During SEEG evaluation, interictal spikes from the right and left hippocampus were seen in runs during sleep, but the spikes from the right hippocampus were more frequent representing 60% of the interictal discharges. All SEEG seizures were localized to the right hippocampus.

**Fig. 1:**
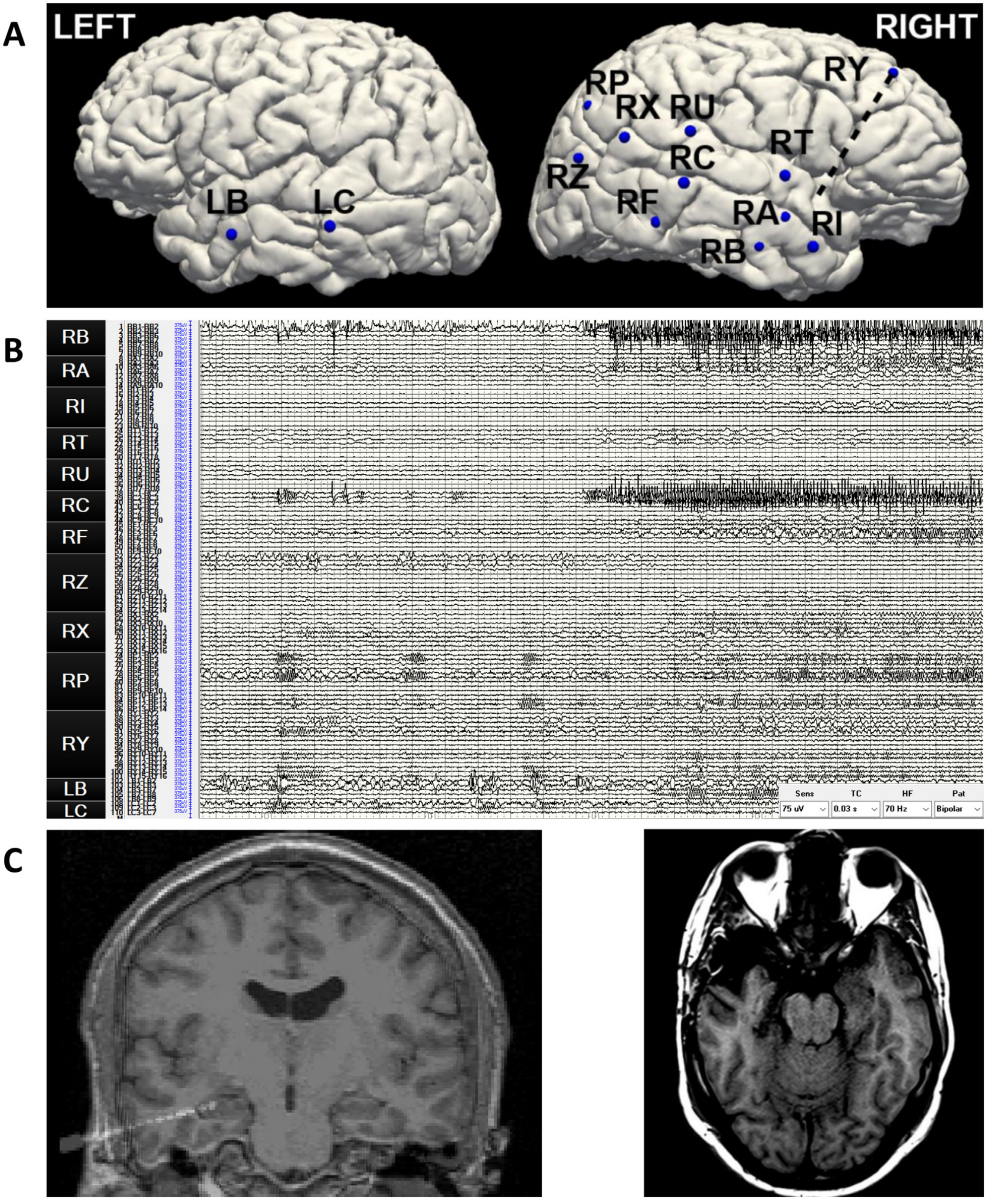
(A) Stereo-electrode coverage sampling limbic and paralimbic network in the right hemisphere, and contralateral mesial and lateral temporal cortex. (B) SEEG ictal onset at RB and RC mesial electrode contacts in the right hippocampus, 30 second page, bipolar montage. (C) Left image: RB electrode sampling the right anterior hippocampus with the mesial contacts. Right image: MRI, post right standard anterior temporal lobectomy.

Two months after the SEEG evaluation, he underwent a standard right anterior temporal lobectomy (ATL) without complications or functional deficit. The surgical pathology was normal. The SEEG evaluation details and post-resection MRI are depicted in Fig. 1.

He was seizure-free for 1 year 6 months after resection. He then reported three FTBC seizures in the subsequent 12 months, although with potential provocation - including significant psychosocial stressors, alcohol consumption and self-decreased doses of ASM.

## 3 Discussion

To our knowledge, this is the first documented report of SEEG evaluation results and resective surgery outcome in an adult patient with epilepsy related to SCN8A mutation. Genetic testing was performed in this patient based on a strong family history of epilepsy, but the initial results of several VUS did not significantly impact surgical-decision making at the time. Only about 3 years after the SEEG evaluation, was the SCN8A mutation reclassified as being likely pathogenic.

The other variants of unknown significance in this patient indicating mutations in deleterious regions of the genes are associated with autosomal recessive epileptic encephalopathies and therefore unlikely to contribute to our patient's epilepsy which segregates in his family in a dominant fashion and is not consistent with a epileptic encephalopathy phenotype.

The optimal role of genetic testing in epilepsy surgery evaluations is still being determined. This case highlights that information obtained from sequencing is dynamic and as the field advances and may later be reclassified [3]. Studies focusing on reclassification rates within specific disease areas have shown reclassification rates of between 6.4 and 15%, but recent report showed only 0.79% of variant reclassifications [6], [7]. Among these reclassifications, majority of VUS variants were reclassified as likely benign or benign (74.6%), compared to 25.4% of VUS variants moving to likely pathogenic or pathogenic [7].

Genetic diagnostics are not routinely performed during presurgical evaluation and reports on epilepsy surgery outcome for patients with genetically-mediated drug-resistant epilepsy are limited, especially in adults [2], [3]. Recent studies have shown that epilepsy surgery may be effective in patients with mutations involving specific genes (mTOR pathway genes as an example), but this has not been demonstrated in patients with other gene mutations [3], [8], [9], [10], [11], [12]. In patients with mutations in genes related to channel function and synaptic transmission, only two patients with SCN1B mutation were seizure-free after temporal lobectomy. The first patient had hippocampal sclerosis (HS), while the second had a non-lesional MRI and normal surgical pathology) [13]. Other patients with drug-resistant focal epilepsy and mutations in SCN1A, CNTNAP2, STBXP1 genes were not seizure-free after epilepsy surgery regardless of lesional status (including HS, focal cortical dysplasia, encephalomalacia present on MRI) [3], [8], [9], [14], [15]. In three patients with sleep-related hypermotor epilepsy phenotype and KCNT1 mutation, epilepsy surgery did not achieve seizure freedom, although epilepsy severity improved in one (Engel class II) [16]. To the best of our knowledge, epilepsy surgery outcomes in patients with SCN8A mutations have not been previously reported.

The SCN8A gene, located on chromosome 12q13.13, is one of the nine human voltage-gated sodium channel genes that is important in the formation of pore-forming alpha subunits of sodium channels in cell membranes and is expressed primarily in the central and peripheral nervous system with minor expression in the membranes of the cardiac muscle fibers [17].

Pathogenic variants in SCN8A have been associated with a wide spectrum of epilepsy phenotypes. They range from benign familial infantile seizures to epileptic encephalopathies and have involved patients with intellectual disability and movement disorders without epilepsy. Several studies reported a favorable seizure response to sodium channels drugs such as carbamazepine, oxcarbazepine, and phenytoin; high doses are often required, and treatment of seizures remains mostly inadequate. Seizures could endure intractable despite partial responses and seizure-free periods [18], [19].

Our patient was heterozygous for SCN8A mutation in exon 16 (c.2671G > A, p.Val891Met). This mutation is located at the pore-forming Na_v_1.6 α-subunit of the sodium channel [20]. The sequence change replaces valine (which is neutral and non-polar) with methionine, which is also neutral and non-polar, at codon 891 of the SCN8A protein (p.Val891Met). This missense change has been observed in other individuals with SCN8A-related conditions [19], [20].

## 4 Conclusion

This case demonstrates that epilepsy surgery may significantly reduce the seizure-burden in a patient with drug-resistant focal epilepsy and a SCN8A mutation. The role of genetic testing in predicting the prognosis for surgical outcomes in epilepsy is still evolving, and further studies on a larger patient cohort are needed to identify the role of epilepsy surgery in patients with drug-resistant epilepsy related to SCN8A mutation. The outcome in this case supports the stance that resective epilepsy surgery should still be an option for people with focal epilepsy associated with a genetic mutation.

## CRediT authorship contribution statement

**Irina Podkorytova:** Conceptualization, Methodology, Validation, Investigation, Resources, Writing – original draft, Writing – review & editing. **Ryan Hays:** Conceptualization, Methodology, Validation, Investigation, Resources, Writing – original draft, Writing – review & editing. **Ghazala Perven:** Methodology, Resources, Writing – review & editing. **Sasha Alick Lindstrom:** Conceptualization, Methodology, Validation, Investigation, Resources, Supervision, Writing – review & editing.

## Declaration of Competing Interest

The authors declare that they have no known competing financial interests or personal relationships that could have appeared to influence the work reported in this paper.
